# Fat Mass Index and Body Mass Index Affect Peak Metabolic Equivalent Negatively during Exercise Test among Children and Adolescents in Taiwan

**DOI:** 10.3390/ijerph15020263

**Published:** 2018-02-04

**Authors:** Shenghui Tuan, Hungtzu Su, Yijen Chen, Minhui Li, Yunjen Tsai, Chunhan Yang, Kolong Lin

**Affiliations:** 1 Department of Rehabilitation Medicine, Cishan Hospital, Ministry of Health and Welfare, No. 60, Zhongxue Rd., Cishan District, Kaohsiung 84247, Taiwan; shtuan@vghks.gov.tw (S.T.); henrydaun.ep96@g2.nctu.edu.tw (H.S.); 2 Department of Physical Medicine and Rehabilitation, Kaohsiung Medical University Hospital, Kaohsiung Medical University, No. 100, Tzyou 1st Road, Kaohsiung 807, Taiwan; 980299@ms.kmuh.org.tw; 3 Department of Physical Medicine and Rehabilitation, Kaohsiung Veterans General Hospital, No. 386, Dazhong 1st Rd., Zuoying District, Kaohsiung 813, Taiwan; mhli@vghks.gov.tw (M.L.); yjtsai@vghks.gov.tw (Y.T.); jsyang@vghks.gov.tw (C.Y.)

**Keywords:** cardiopulmonary fitness, peak metabolic equivalent, fat mass index, body mass index, childhood obesity

## Abstract

Peak metabolic equivalent (MET) is the most reliable indicator of cardiorespiratory fitness (CRF). The aim of this study was to examine the association between CRF indicated by peak MET and body mass index (BMI) or fat mass index (FMI) in Taiwanese children and adolescents (C-A). Data of 638 C-A aged 10–18 that received symptom-limited treadmill exercise testing was analyzed. Anthropometry-body composition was measured by vector bioelectrical impedance analysis. BMI was defined as body weight (kg)/body height (m)^2^ and FMI was defined as fat mass (kg)/body height (m)^2^. BMI was grouped by Taiwanese obesity cut-off points. FMI Class-I was categorized by percentage of body fat. FMI Class-II used the reference values from Korean C-A. Excess adiposity was defined as (1) “overweight” and “obesity” by BMI, (2) greater than the sex- and age-specific 75th percentile of whole subjects by FMI Class-I, and (3) greater than 95th percentiles of reference value by FMI Class-II. Boys had significantly higher fat mass and FMI, and had more excess adiposity than girls (all *p* < 0.05). Both boys and girls with excess adiposity (by any definition) had lower MET at anaerobic threshold (AT MET) and peak MET (all *p* < 0.001). BMI and FMI were significantly negatively associated with both AT MET and peak MET significantly (all *p* < 0.001). FMI (95% CI: −0.411~−0.548) correlated with peak MET more than BMI (95% CI: −0.134~ −0.372) did. Excess adiposity affected CRF negatively. It is concluded that weight management should start early in childhood.

## 1. Introduction

Childhood obesity is an important issue of public health now. It can cause many adverse health consequences, such as cardiovascular (CV) disease and type 2 diabetes mellitus [1]. Childhood obesity might persist into adulthood and is associated with increased morbidity and mortality [2]. The prevalence of childhood obesity has been rising worldwide over the last few decades [3]. In Taiwan, according to a nationwide survey by Ministry of Education in 2003, the prevalence of overweight and obesity was 25.2% among school boys and 15.2% among school girls [4].

Higher body mass index (BMI) and increased percentage of fat mass (%FM) have been shown to be negatively related with cardiopulmonary fitness (CRF) level in children and adolescents in both normal weight or overweight populations [5,6,7]. Many previous studies have shown that obese children or adolescents have lower CRF [8,9]. A lower CRF also has negative effects on the CV system [9].

Since BMI reflects both the fat mass (FM) and the fat-free mass (FFM) in the body, it might be poorly correlated with %FM in children and adolescents [10]. Studies have also shown that FM index (FMI), defined as FM (kg) divided by height squared (m^2^), better discriminates adiposity compared to BMI [11] and %FM [12]. Studies are showing now that a higher FMI, like a higher BMI and increased %FM, is associated with CV diseases [13] and metabolic syndrome [14]. There are many ways to measure body composition, and such efforts can be divided into simple measurements or indices, such as measurements of skinfold thickness and waist circumference, densitometry or dual energy X-ray absorptiometry (DEXA), and predictive techniques, such as bioelectric impedance analysis (BIA). Each have their own advantages and disadvantages [15]. BIA is easier and cheaper to perform than densitometry and DEXA and, compared to skinfold thickness and waist circumference measurements, more accurately determines body fat. Body composition measurements in children and adolescents are inherently challenging, because of the rapid growth-related changes in height, weight, FFM and FM. One recent and large systemic review has shown that body fat percentage estimated by BIA exhibited almost perfect reproducibility, and FM and FFM estimated by BIA correlated almost perfectly with the reference methods in both sexes in children and adolescents [16].

CRF is defined as the overall capacity of the cardiovascular and respiratory systems to carry out prolonged strenuous exercise. Many studies have assessed the CRF of children and adolescents using established indirect measurements, such as 800 m runs [7], 20 m multistage fitness tests [17], and progressive aerobic cardiovascular endurance runs [6], instead of direct exercising tests. Few studies assessed CRF by a cycle ergometer [18]. Though these measurements have been established to be in good correlation to CRF, maximum oxygen uptake (VO_2_ max) attained during a graded exercise testing to voluntary exhaustion is generally considered to be the gold standard for determining CRF and aerobic fitness [19].

To the best of our knowledge, this is the first study to investigate associations between FMI and CRF attained via treadmill exercising tests in Chinese children and adolescents. The main objective of this study was to examine the relationship between FMI and CRF assessed by peak oxygen uptake (peak O_2_) during treadmill exercise testing. Since (1) BMI remains a simple and easy measure to use in most settings, and there is a well-established reference value for classifying obesity or not in Taiwan, and (2) there are no available reference values of FIM for Taiwan children and adolescents currently, we assessed the association between BMI and CRF in this study.

## 2. Materials and Methods

### 2.1. Subject Characteristics

The study was conducted at Kaohsiung Veterans General Hospital, Taiwan, from February 2011 to March 2017. All children and adolescents (aged from 10 to 18 years old) without known significant medical conditions and detectable cardiovascular disease (examined by 12-lead electrocardiogram and transthoracic echocardiographic examination) were recruited randomly. Most of them were from the city of Kaohsiung in Southern Taiwan and visited our outpatient clinic for detailed examination after primary health screening at schools. Before enrollment, each participant was familiarized with the procedures and equipment used in the treadmill exercise testing through a demonstrative explanation. The purpose of the study was explained to the subjects and their families before informed written consent was obtained. This study was conducted in accordance with the Helsinki Declaration and was approved by the Institutional Review Board of Kaohsiung Veterans General Hospital (number: VGHKS15-CT7-05).

### 2.2. Treadmill Exercise Testing

We used graded symptom-limited exercise testing, which involved a treadmill, a flow module, a gas analyzer, and an electrocardiographic monitor (Metamax 3B, Cortex Biophysik GmbH Co., Leipzig, Germany), to measure the subjects’ exercise capacity. All subjects underwent the testing according to the Bruce ramp protocol suggested by American College of Sports Medicine. We terminated the test when the subjects demonstrated subjective unbearable symptoms or when they could no longer continue [20]. We measured metabolic equivalent (MET), blood pressure (BP), and heart rate (HR) throughout the testing. We recorded MET at anaerobic threshold (AT MET). The peak O_2_ was determined as a failure of oxygen uptake to increase by greater than 2.0 mL kg^−1^ min^−1^ with treadmill speed/inclination increase. Peak MET was calculated as peak O_2_ divided by 3.5 mL kg^−1^ min^−1^.

### 2.3. Anthropometry-Body Composition

Height and weight of barefooted subjects in light clothing were measured during visit. All measurements were taken by a trained physical therapist following standard operating procedures. Anthropometry-body composition was measured by vector bioelectrical impedance analysis (VBIA), which is a useful tool for body composition analysis in healthy adults and children. The VBIA was performed with bioelectrical impedance vector analysis software by the resistance-reactance graph method [10]. To analyze the body composition of our subjects, we used Zeus 9.9 PLUS (Jawon Medical Co., Ltd., Kungsang Bukdo, Korea), which sent a minute electric current and measured the body composition using personal data that had already been saved (height, weight, sex, age, and newly calculated body impedance) by the Tetrapolar electrode method (electrodes were located on both hands, both soles of the feet, and both ankles of subjects, with frequencies of 1, 5, 50, 250, 550, and 1000 kHz and a 360 μA current).

BMI was calculated by dividing weight by the square of the subjects’ height. Children and adolescents were categorized as “underweight,” “normal weight,” “overweight,” and “obese” using standard age- and gender-specific BMI values published in 2013 by Ministry of Education of Taiwan (http://www.fitness.org.tw/model08.php) [21]. FMI was defined as FM (kg) divided by squared (m^2^) of the subjects’ height and FFMI was defined as FFM (kg) divided by squared (m^2^) of the subjects’ height.

Since there were no available reference values of FIM for Taiwan children and adolescents before the study published, we chose two systems to classify FMI. The 75th–85th percentile for percentage of body fat (%BF) has been shown to correspond with excess adiposity in children and adolescents [22], and the 75th percentile for %BF has been used as the criteria for identifying excess adiposity in a study of dyslipidemia [23]. Given this precedent, we defined excess adiposity as an FMI greater than the sex- and age-specific 75th percentile, insufficient adiposity as an FMI below the 5th percentile, and normal adiposity as an FMI between the 5th and 75th percentiles, as per the suggestion of Weber DR et al. [24] (defined as FMI Class-I). We used the reference values of FMI for Korean children and adolescents [25] and classified our subjects into 2 groups based on the percentiles of the nationwide sample: normal adiposity: ≤95th percentile; excess adiposity: >95th percentiles [26] (defined as FMI Class-II).

### 2.4. Pulmonary Function Test

To avoid cofounding factors in the correlation analysis, a pulmonary function test was performed by the spirometry at rest to obtain control variables. Forced vital capacity (FVC) and forced expiratory volume in one second (FEV1) were measured.

### 2.5. Statistical Analysis

We used SPSS for Windows version 19.0 (Released 2010. IBM Corp., Armonk, NY, USA) for all analyses. Descriptive statistics for gender, height, weight, BMI, FM, FMI, FFM, FFMI, and percentage of excess adiposity were calculated to characterize the participants. Normality and homoscedasticity were checked before each analysis. All participants were analyzed with respect to gender difference in height, weight, BMI, FM, FMI, FFM, FFMI, and percentage of excess adiposity (by BMI classification and FMI Class-II) with independent sample *t*-tests for normally distributed variables and chi-square tests for differences in the distribution between categorized variables. For CRF analysis, we used independent sample *t*-tests to compare the normal adiposity group with the excess adiposity group using three different definitions (BMI, FMI Class-I, and FMI Class-II). We performed a multiple stepwise regression, performed with AT MET or peak MET as dependent variables, and with baseline pulmonary function (including FVC and FEV1) and age as the predictor variables. The associations between BMI, FMI, and CRF (AT MET and peak MET) of all subjects were examined using Pearson’s simple correlation analysis and partial correlation analysis. Partial correlation analysis was used to avoid statistical confounding. A *p*-value ≤ 0.05 was considered statistically significant.

## 3. Results

Six hundred and forty-one subjects were recruited. Among them, three did not complete the treadmill exercise testing (one due to shortness of breath and two due to leg soreness). Therefore, the final data we analyzed was from 638 subjects. Table 1 summarizes the baseline characteristics of 638 subjects (boys = 302, girls = 336) we analyzed at the end. The prevalence of overall combined overweight and obese children by BMI and FMI, Class-II definition, was 22.1% and 16.2%, respectively. Boys had significantly higher FM, FMI, FFM, and FFMI than girls (all *p* < 0.05). Boys were significantly more overweight/obese (28.8% vs. 18.0%, *p* < 0.001) and had more excess adiposity (19.7% vs. 13.1%, *p* = 0.024) than girls.

Table 2 summarizes the baseline characteristics of all subjects of different ages (one year per subgroup, from 10 to 18 years old). Boys had significantly higher FFM and FFMI than girls in all 9 subgroups. Boys had significantly higher FM and FMI than girls, but there were significant differences in FMI only in subgroups of 14–18 years old. Boys were significantly more overweight/obese by BMI definition in all subgroups, but there was a significant difference only in subgroups of 11 and 17 years old (*p* = 0.022 and 0.021, respectively). Boys had more excess adiposity than girls but only had significance in the subgroup of 17 years old (*p* = 0.008).

Table 3 shows the results of comparisons of CRF between subjects of excess and normal adiposity by BMI and FMI, Class-I and Class-II definitions. Both boys and girls with normal adiposity had higher AT MET and peak MET by all definitions (all *p* < 0.001) and had higher HRR by FMI, Class-I definition, (*p* = 0.007) than those with excess adiposity. In respect of gender, girls with normal adiposity had higher AT MET and peak MET by FMI, Class-I and Class-II definitions, (all *p* < 0.01), and higher peak MET by the BMI definition (*p* = 0.001) than those with excess adiposity. Boys with normal adiposity had higher AT MET and peak MET by all definitions (all *p* < 0.001) than those with excess adiposity.

In analysis of the relationship between body fat index (BMI or FMI) and AT MET or peak MET, stepwise regression analysis revealed only one model that accounted for substantial proportions of AT MET or peak MET of all the subjects. In each model, BMI or FMI was the only predictor, and all other variables (FVC, FEV1, and age) were excluded from the model. Table 4 demonstrated the Pearson’s simple correlations and partial correlation (using FVC, FEV1, and age as control variables) between variables of exercise capacity (AT MET and peak MET), BMI, and FMI. BMI was significantly negatively associated with both AT MET and peak MET. The Pearson’s correlation coefficient was −0.172 (95% CI: −0.101~−0.317, *p* < 0.001) and −0.207 (95% CI: −0.134~−0.372, *p* < 0.001), respectively. Both showed modest correlations. FMI was significantly negatively associated with AT MET and peak MET. The Pearson’s correlation coefficient was −0.400 (95% CI: −0.310~−0.449, *p* < 0.001), and −0.471 (95% CI: −0.411~−0.548, *p* < 0.001), respectively. Both showed modest to moderate correlations. The results of the partial correlation analysis revealed (1) that the BMI was positively correlated with AT MET (correlation coefficient = −0.106, *p* < 0.01) and peak MET (correlation coefficient = −0.137, *p* < 0.01) and (2) that the FMI was positively correlated with AT MET (correlation coefficient= −0.334, *p* < 0.01) and peak MET (correlation coefficient = −0.393, *p* < 0.01).

## 4. Discussion

Our study investigated the relationship between BMI, FMI, and CRF assessed by peak oxygen uptake (peak O_2_) during treadmill exercise testing in Taiwanese children and adolescents aged from 10 to 18 years old. Although the childhood obesity trend in Southern Taiwan is generally higher than in other regions of Taiwan according to the study of Chu et al. in 2007 [27], our results showed the prevalence of overweight and obese children for both boys and girls (boys: 28.8%; girls 18.0%) was similar to that found in the national survey in Taiwan (boys: 29%; girls: 21%) [28]. The definition of excess adiposity we used for FMI Class-II was FMI > 95th of the Korean reference values and is consistent with the definition of fat defined by percentile [26]. Using the FMI Class-II definition, the prevalence of fat in both genders (boys: 19.7%; girls: 13.1%) was much higher than that defined by the BMI (boys: 13.2%; girls: 7.1%). This result corresponds with a previous study showing that FMI better discriminates adiposity than BMI [11]. We used Korean reference values due to a lack of available FMI data among Taiwanese children and adolescents, but the FMI of both girls and boys in our study was much lower than those of the Korean subjects in each subgroup classified by age. To clarify this difference, further larger and nationwide studies are warranted to provide FMI reference values among Taiwanese children and adolescents.

Just as previous studies have shown, boys had a significantly higher fat mass, FMI, fat-free mass, FFMI, and CRF than girls in our study [29,30]. These differences in body composition and CRF might result from differences in physiological characteristics, the onset of puberty, and the physical developmental factors during the adolescence period. Furthermore, the differences in behavioral characteristics and environmental risk factors between boys and girls might also affect the association between CRF and childhood obesity [17]. However, since these differences might be multifactorial, there is no consensus on the gender-specific effects of CRF or childhood obesity [17,29,30]. This study focused on the relationship between body fat and CRF. We did not make further investigations on, for example, the activity level involved in daily life, the environmental barrier or access to physical activity, the onset of puberty, or the current status of puberty level of our subjects. We also could not speculate which factor contributed to the differences in CRF between genders more.

CRF has been shown to be a better index of the activity level than direct and short-term measures of physical activity [31]. Many previous studies from different countries have demonstrated that obese (by BMI definition) children or adolescents have lower CRF [8,9]. He et al. [17] and Hsieh et al. [7] found a relatively strong negative association between CRF levels and BMI of Chinese and Taiwanese children. Studies evaluating the association between FMI and CRF are much less common than those studying the association between BMI and CRF. A few studies have showed that lower FMI was associated with better CRF [32]. Our results are similar to those of these studies and indicate that, regardless of which BMI or FMI classifications are considered, Taiwanese children and adolescent with normal adiposity have better CRF than those with excess adiposity. However, it has been shown that maturation has a significantly positive effect on aerobic performance among children and adolescents aged 6–14 [33,34]. To exclude the cofounding factors that might influence of CRF, we performed a multiple stepwise regression analysis using BMI or FMI, age, and baseline pulmonary function as predicting variables and found that BMI or FMI still contributed the most to CRF in each model. Partial correlation analysis using age as control variables also showed significant negative relationship between CRF and BMI or FMI, even though the correlation coefficient of each was relatively smaller. Therefore, our study indicated that BMI and FMI are both independent factors in determining AT MET and peak MET. In addition, in the two-variable correlation analysis, we found that the 95% confidence interval of AT MET and BMI (−0.101~−0.327) overlapped with that of AT MET and FMI (−0.310~−0.449). Whereas the 95% confidence interval of the peak MET and BMI (−0.134~−0.372) was behind that of the peak MET and FMI (−0.411~−0.548). Therefore, we could state that FMI correlates with peak MET more than BMI did.

The uniqueness of our study was that we measured the CRF of children and adolescents directly by graded symptom-limited treadmill exercise testing with a gas analyzer rather than other indirect measurements such as 800 m runs [7] and 20 m multistage fitness tests [17] that have been used previously to reflect the CRF of Taiwanese or Chinese children. Directly acquiring the peak O_2_ might have led to the more accurate reflections of CRF in our subjects. Furthermore, this is the first study to examine the relationship between FMI and CRF assessed by peak O_2_ among Chinese children and adolescents. FMI is a well-established indicator of fat, and many studies have shown that it is better to discriminate adiposity than BMI.

Despite its contributions, our study has several limitations. First, due to its cross-sectional design, the results regarding the relationship between body fat and CRF levels in Taiwanese children should be interpreted carefully. Conclusions on the direction of the relationships cannot be drawn. Second, the subjects were recruited randomly in a single medical center in Southern Taiwan, so the results might be only generalizable to similar populations, even though the prevalence of overweight and obese children was similar to data found in the national survey in Taiwan [28]. Finally, due to a lack of available surveys of normal distribution of FMI data among Taiwanese children and adolescents, the reference of excess adiposity we used for FMI Class-II was from Korean population. Although the weight and height were similar between Taiwanese and Korean children and adolescents [21,35], the body composition might be different. Further large-scale and nationwide studies are warranted to provide reference values of FMI among Taiwanese children and adolescents to clarify this difference.

## 5. Conclusions

Our study showed a negative association between body adiposity and CRF levels among Taiwanese children and adolescents, regardless of which definitions of BMI and FMI are used. Boys and young men performed significantly better on CRF tests than girls and young women did. Given the high prevalence of excess adiposity in children and adolescents, weight control and health promotion are important for improving public health. FMI correlates with peak MET more than BMI does. Further studies should be conducted to provide FMI reference data and to assess FIM as an early detector of childhood obesity for earlier positive reinforcement in Taiwanese children and adolescents. Larger prospective studies are warranted to elucidate the complex relationships between CRF level and body adiposity.

## Figures and Tables

**Table 1:** Baseline characteristics of all subjects aged from 10 to 18 years old.

		Height (cm)	Weight (kg)	BMI (kg/m^2^)	U (%)	N (%)	O (%)	F (%)	FM (kg)	FMI (kg/m^2^)	FFM (kg)	FFMI (kg/m^2^)	Excess Adiposity by FMI II
Girl	N = 336	152.50 ± 10.44	46.14 ± 11.20	19.54 ± 3.76	18.8	65.2	8.9	7.1	11.01 ± 5.72	4.68 ± 2.28	35.13 ± 6.41	14.99 ± 1.60	13.1%
Boy	N = 302	160.64 ± 13.41	54.09 ± 17.19	20.91 ± 7.49	17.2	54.0	15.6	13.2	9.84 ± 8.26	3.69 ± 2.80	44.25 ± 10.23	16.93 ± 2.10	19.7%
Total	N = 638	156.35 ± 12.60	49.90 ± 14.88	20.19 ± 5.86	18.0	59.9	12.1	10.0	10.46 ± 7.05	4.21 ± 2.58	39.44 ± 9.58	15.91 ± 2.29	16.2%
*p*-value		<0.001 *	<0.001 *	0.004 *	<0.001 *^,a^				0.040 *	<0.001 *	<0.001 *	<0.001 *	0.024 *^,a^

BMI: body mass index; U (%): percentage of underweight subjects; N (%): percentage of normal weight subjects; O (%): percentage of overweight subjects; F (%): percentage of fat subjects; FM: fat mass; FMI: fat mass index; FFM: fat-free mass; FFMI: fat-free mass index; Excess adiposity by FMI II: >95th percentile of FIM according to the reference values of Korean. ^a^ All the comparisons between girls and boys were done by independent *t*-test except *p*-values marked with a, which were analyzed by an independent chi square test for comparison percentage of excess adiposity between girls and boys; * *p*-value < 0.05.

**Table 2:** Baseline characteristics of all subjects by age- and sex-specific classifications.

Age	Girl	Height (cm)	Weight (kg)	BMI (kg/m^2^)	U (%)	N (%)	O (%)	F (%)	FM (kg)	FMI (kg/m^2^)	FFM (kg)	FFMI (kg/m^2^)	Excess Adiposity by FMI Class-II
	Boy												
	Total												
10	N = 58	138.56 ± 8.62	35.40 ± 8.40	18.34 ± 3.47	13.8	65.5	8.6	12.1	7.74 ± 4.20	4.04 ± 2.09	27.65 ± 5.02	14.30 ± 1.56	13.8%
	N = 35	141.08 ± 6.14	39.80 ± 10.90	19.79 ±4.10	8.6	60.0	20.0	11.4	7.06 ± 5.89	3.42 ± 2.53	32.75 ± 5.31	16.38 ± 1.70	17.1%
	N = 93	139.51 ± 7.84	37.05 ± 9.60	18.88 ± 3.76	11.8	63.4	12.9	11.8	7.48 ± 4.88	3.81 ± 2.27	29.57 ± 5.68	15.08 ± 1.90	15.1%
*p*-Value		0.135	0.031 *	0.070	0.245 ^a^				0.514	0.207	<0.001 *	<0.001 *	0.662 ^a^
11	N = 39	146.70 ± 9.87	37.30 ± 7.72	17.25 ± 2.82	41.0	51.3	5.1	2.6	7.09 ± 3.63	3.33 ± 1.81	30.20 ± 5.25	13.94 ± 1.21	7.7%
	N = 32	145.94 ± 7.72	40.03 ± 11.44	18.78 ± 4.22	25.0	46.9	9.4	18.8	6.52 ± 5.32	2.99 ± 2.29	33.52 ± 6.99	15.61 ± 2.32	3.1%
	N = 71	146.36 ± 8.97	38.53 ± 9.60	17.94 ± 3.58	33.8	49.3	7.0	9.9	6.83 ± 4.45	3.17 ± 2.03	31.70 ± 6.28	14.69 ± 1.97	5.6%
*p*-Value		0.725	0.253	0.085	0.022 *^,a^				0.605	0.488	0.026 *	<0.001 *	0.406 ^a^
12	N = 38	151.02 ± 9.07	43.86 ± 8.28	18.62 ± 3.90	7.9	78.9	10.5	2.6	9.19 ± 4.18	4.07 ± 1.95	34.67 ± 5.52	15.17 ± 2.05	10.5%
	N = 50	153.86 ± 8.65	48.29 ± 11.75	22.38 ± 14.86	12.0	58.0	16.0	14.0	8.21 ± 5.95	3.44 ± 2.40	40.08 ± 6.90	16.84 ± 1.96	18.0%
	N = 88	152.63 ± 8.90	46.38 ± 10.57	20.76 ± 11.59	10.2	67.0	13.6	9.1	8.63 ± 5.26	3.71 ± 2.23	37.75 ± 6.86	16.12 ± 2.16	14.8%
*p*-Value		0.138	0.041 *	0.132	0.084 ^a^				0.388	0.192	<0.001 *	<0.001 *	0.436 ^a^
13	N = 36	157.02 ± 7.29	48.64 ± 9.67	19.70 ± 3.56	25.0	55.6	13.9	5.6	11.38 ± 5.42	4.61 ± 2.24	37.26 ± 5.19	15.07 ± 1.49	16.7%
	N = 43	163.20 ± 8.65	55.81 ± 12.10	20.38 ± 4.73	9.3	58.1	18.6	14.0	9.64 ± 7.03	3.59 ± 2.49	46.16 ± 6.00	17.31 ± 1.57	15.0%
	N = 79	160.39 ± 8.59	52.54 ± 11.57	20.07 ± 4.22	16.5	57.0	16.5	10.1	10.43 ± 6.37	4.06 ± 2.42	42.11 ± 7.17	16.29 ± 1.89	15.8%
*p*-Value		0.001 *	0.005 *	0.477	0.189 ^a^				0.230	0.061	<0.001 *	<0.001 *	0.842 ^a^
14	N = 40	157.46 ± 5.60	51.60 ± 11.62	20.47 ± 4.83	22.5	60.0	5.0	12.5	13.43 ± 7.38	5.42 ± 2.92	38.17 ± 4.90	15.37 ± 1.65	17.5%
	N = 38	166.50 ± 8.66	58.82 ± 19.21	20.80 ± 6.46	26.3	474	13.2	13.2	11.45 ± 11.31	4.05 ± 3.93	47.37 ± 9.03	16.99 ± 2.40	23.7%
	N = 78	161.87 ± 8.52	55.12 ± 16.09	20.63 ± 5.64	24.4	53.8	9.0	12.8	12.47 ± 9.49	4.75 ± 3.49	42.65 ± 8.53	16.16 ± 2.19	20.5%
*p*-Value		<0.001 *	0.050 *	0.794	0.346 ^a^				0.361	0.085	<0.001 *	<0.001 *	0.499 ^a^
15	N = 49	158.52 ± 5.43	53.36 ± 9.25	21.23 ± 3.50	8.2	67.3	18.4	6.1	14.15 ± 5.84	5.64 ± 2.31	39.21 ± 4.17	15.60 ± 1.37	20.4%
	N = 43	169.40 ± 7.14	61.82 ± 18.52	21.61 ± 4.72	27.9	39.5	20.9	11.6	11.74 ± 9.47	3.99 ± 2.93	50.07 ± 9.90	17.38 ± 2.61	34.9%
	N = 92	163.61 ± 8.30	57.31 ± 14.89	21.41 ± 4.10	17.4	54.3	19.6	8.7	13.02 ± 7.80	4.87 ± 2.73	44.29 ± 9.17	16.41 ± 2.22	27.2%
*p*-Value		<0.001 *	0.006 *	0.663	0.391 ^a^				0.141	0.003 *	<0.001 *	<0.001 *	0.119 ^a^
16	N = 30	157.30 ± 6.14	50.38 ± 7.12	20.24 ± 2.98	20.0	66.7	6.7	6.7	12.50 ± 4.40	5.08 ± 1.82	37.89 ± 3.27	15.34 ± 1.39	10.0%
	N = 33	172.33 ± 5.96	62.34 ± 13.29	20.96 ± 4.38	12.1	66.7	15.2	6.1	11.08 ± 8.10	3.73 ± 2.71	51.27 ± 5.62	17.27 ± 1.77	18.2%
	N = 63	165.18 ± 9.65	58.65 ± 12.30	20.61 ± 3.76	15.9	66.7	11.1	6.3	11.75 ± 6.59	4.37 ± 2.41	44.89 ± 8.16	16.35 ± 1.86	14.3%
*p*-Value		<0.001 *	<0.001 *	0.452	0.411 ^a^				0.398	<0.001 *	0.025 *	<0.001 *	0.354 ^a^
17	N = 38	158.88 ± 5.09	52.24 ± 8.64	20.68 ± 3.26	15.8	73.7	2.6	7.9	13.35 ± 5.30	5.30 ± 2.09	38.89 ± 4.15	15.40 ± 1.35	7.9%
	N = 23	175.19 ± 4.88	70.06 ± 17.97	22.67 ± 4.94	17.4	47.8	13.0	21.7	14.94 ± 10.40	4.78 ± 3.13	55.12 ± 7.76	17.90 ± 1.86	34.8%
	N = 61	165.03 ± 9.40	58.96 ± 15.50	21.43 ± 4.05	16.4	63.9	6.6	13.1	13.95 ± 7.59	5.10 ± 2.52	45.01 ± 9.78	16.34 ± 1.97	18.0%
*p*-Value		<0.001 *	<0.001 *	0.094	0.021 *^,a^				0.433	0.440	<0.001 *	<0.001 *	0.008 *^,a^
18	N = 8	158.42 ± 6.47	50.35 ± 6.33	20.06 ± 1.78	25.0	75.0	0	0	12.89 ± 2.87	5.13 ± 1.07	37.46 ± 4.03	14.90 ± 0.86	0%
	N = 6	170.45 ± 6.80	58.18 ± 8.58	19.93 ± 1.60	16.7	83.3	0	0	10.00 ± 3.76	3.40 ± 1.08	48.18 ± 5.24	16.54 ± 0.62	0%
	N = 14	163.58 ± 8.86	53.71 ± 8.13	20.01 ± 1.64	21.4	78.6	0	0	11.65 ± 3.47	4.39 ± 1.36	42.06 ± 7.04	15.60 ± 1.12	0%
*p*-Value		0.006 *	0.072	0.891	NA				0.128	0.011 *	0.001 *	0.002 *	NA

BMI: body mass index; U (%): percentage of underweight subjects; N (%): percentage of normal weight subjects; O (%): percentage of overweight subjects; F (%): percentage of fat subjects; FM: fat mass; FMI: fat mass index; FFM: fat-free mass; FFMI: fat-free mass index; Excess adiposity by FMI Class-II: >95th percentile of FIM according to the reference values of Korean; NA: not applicable. Data are present as mean (SD), or percentage when appropriate. ^a^ All the comparisons between girls and boys were done by independent *t*-test except *p*-values marked with a, which were analyzed by an independent chi square test for comparison percentage of excess adiposity between girls and boys; * *p*-value < 0.05.

**Table 3:** Cardiopulmonary function between subjects of excess and normal adiposity under different classifications.

Classify by BMI	Girl			Boy			Total		
	N (N = 217)	E (N = 54)	*p*-Value	N (N = 163)	E (N = 87)	*p*-Value	N (N = 380)	E (N = 141)	*p*-Value
AT MET	6.16 ± 1.09	5.90 ± 1.10	0.129	7.41 ± 1.25	6.33 ± 1.35	<0.001 *	6.70 ± 1.32	6.17 ± 1.27	<0.001 *
PEAK MET	8.71 ± 1.50	7.98 ± 1.44	0.001 *	10.93 ± 1.69	9.09 ± 2.05	<0.001 *	9.66 ± 1.92	8.67 ± 1.91	<0.001 *
Classify by FMI Class-I	N (N = 240)	E (N = 81)	*p*-value	N (N = 219)	E (N = 72)	*p*-value	N (N = 459)	E (N = 153)	*p*-value
AT MET	6.30 ± 1.10	5.88 ± 1.15	0.003 *	7.42 ± 1.30	6.10 ± 1.22	<0.001 *	6.84 ± 1.34	5.98 ± 1.18	<0.001 *
PEAK MET	8.92 ± 1.48	8.01 ± 1.37	<0.001 *	10.90 ± 1.73	8.81 ± 2.02	<0.001 *	9.86 ± 1.88	8.38 ± 1.75	<0.001 *
Classify by FMI Class-II	N (N = 290)	E (N = 44)	*p*-value	N (N = 240)	E (N = 59)	*p*-value	N (N = 530)	E (N = 103)	*p*-value
AT MET	6.24 ± 1.10	5.77 ± 1.14	0.009 *	7.35 ± 1.33	6.07 ± 1.27	<0.001 *	6.75 ± 1.34	5.94 ± 1.22	<0.001 *
PEAK MET	8.80 ± 1.48	7.85 ± 1.42	<0.001 *	10.77 ± 1.78	8.78 ± 2.10	<0.001 *	9.69 ± 1.90	8.38 ± 1.89	<0.001 *

BMI: body mass index; FMI: fat mass index; N: normal adiposity; E: excess adiposity; AT MET: metabolic equivalent at anaerobic threshold; PEAK MET: peak metabolic equivalent during exercise testing; Classify by BMI: a normal BMI was defined as normal adiposity, while an overweight or obese BMI was defined as excess adiposity; Classify by FMI Class-I: an FMI ≥5th percentile but ≤75th percentile of all the subjects was defined as normal adiposity, while an FMI >75th percentile of all the subjects was defined as excess adiposity; Classify by FMI Class-II: an FMI ≤95th percentile of the reference values of Korean children and adolescents was defined as normal adiposity, while an FMI >95th percentile of the reference values of Korean children and adolescents was defined as excess adiposity; * *p*-value < 0.05.

**Table 4:** Correlation between body mass index, fat mass index, and performance of exercise test of children and adolescents.

	Simple Correlation		Partial Correlation	
	AT MET	PEAK MET	AT MET	PEAK MET
BMI	−0.172 (95% CI: −0.101~−0.327)	−0.207 (95% CI: −0.134~−0.372)	−0.106	−0.137
	<0.001 *	<0.001 *	<0.001 *	<0.001 *
FMI	−0.400 (95% CI: −0.310~−0.449)	−0.474 (95% CI: −0.411~−0.548)	−0.334	−0.393
	<0.001 *	<0.001 *	<0.001 *	<0.001 *

BMI: body mass index; FMI: fat mass index; AT MET: metabolic equivalent at anaerobic threshold; PEAK MET: peak metabolic equivalent during exercise testing; CI: confidence interval; Upper row: Pearson’s coefficient factor; Lower row: *p*-value; * *p*-value < 0.001.
